# Sugar-sweetened beverage intake and serum testosterone levels in adult males 20–39 years old in the United States

**DOI:** 10.1186/s12958-018-0378-2

**Published:** 2018-06-23

**Authors:** Liang Chen, Yu-Mei Xie, Jian-Hao Pei, Jian Kuang, Hong-Mei Chen, Zhong Chen, Zhong-Wen Li, Xiao-Ying Fu, Long Wang, Shui-Qing Lai, Shu-Ting Zhang, Zhi-Jiang Chen, Jin-xin Lin

**Affiliations:** 1 The First Division in the Department of Endocrinology, Guangdong General Hospital, Guangdong Academy of Medical Sciences, 106th of Zhongshan Er Road, Guangzhou, 510080 China; 2 GuangDong Cardiovascular Institute, Guangdong General Hospital, Guangdong Academy of Medical Sciences, Guangzhou, China

**Keywords:** Hypogonadism, National Health and nutrition examination survey (NHANES), Sugar-sweetened beverages (SSBs), Testosterone

## Abstract

**Background:**

This population-based study was designed to investigate whether consumption of sugar-sweetened beverages (SSB) is associated with lower serum total testosterone concentration in men 20–39 years old.

**Methods:**

All data for this study were retrieved from the National Health and Nutrition Examination Survey (NHANES) 2011–2012. The primary outcome was serum testosterone concentration, and main independent variable was SSB intake. Other variables included age, race/ethnicity, poverty/income ratio, body mass index (BMI), serum cotinine, heavy drinking, and physical activity.

**Results:**

Among all subjects (*N* = 545), 486 (90.4%) had normal testosterone levels (defined as ≥231 ng/dL) and 59 (9.6%) had low testosterone levels (defined as < 231 ng/dL). Multivariate logistic regression revealed the odds of low testosterone was significantly greater with increasing SSB consumption (Q4 [≥442 kcal/day] vs. Q1 [≤137 kcal/day]), adjusted odds ratio [aOR] = 2.29, *p* = 0.041]. After adjusting for possible confounding variables, BMI was an independent risk factor for low testosterone level; subjects with BMI ≥ 25 kg/m^2^ had a higher risk of having a low testosterone level than those with BMI < 25 kg/m^2^ (aOR = 3.68, *p* = 0.044).

**Conclusion:**

SSB consumption is significantly associated with low serum testosterone in men 20–39 years old in the United States.

**Electronic supplementary material:**

The online version of this article (10.1186/s12958-018-0378-2) contains supplementary material, which is available to authorized users.

## Background

Male hypogonadism is a condition in which the body produces insufficient testosterone, the male reproductive hormone that supports masculine growth and development during puberty and enables adult males to produce sperm and reproduce [1]. Reproductive functions may be impaired when total testosterone concentrations are reduced [1]. A study of serum total testosterone concentrations in household populations participating in the National Health and Nutrition Examination Survey (NHANES) in 2011–2012 revealed different patterns of testosterone level between age groups, and between race/ethnic groups. Higher testosterone levels were found in non-diabetic males compared to those with diabetes, and in more physically active men than sedentary men [2]. The authors of that study suggested that these differences may be associated with testosterone metabolism, or with different health outcomes.

Several studies using the NHANES database have reported multiple health effects of consuming sugar-sweetened beverages (SSBs), including weight gain, type 2 diabetes, cardiovascular disease, hyperuricemia, and gout [3–5]. Sugar-sweetened beverage consumption is the main source of added sugar in the diet of people in the United States, and is reported to contribute significantly to weight gain and increased risk of diabetes and cardiovascular disease [6]. Consuming fructose from any sugar, and high-fructose corn syrup in large quantities contributes to a high dietary glycemic load that leads to insulin resistance, inflammation, and increased risk of metabolic and cardiovascular disease [7]. Cardiometabolic risk factors have been identified in adolescents and adults who consume SSBs [8, 9].

Some findings suggest consumption of SSBs may impact fertility. Machtinger et al. (2017) reported that SSBs may reduce the number and quality of oocytes and number of cycles resulting in live births in women undergoing in vitro fertilization [10]. The immediate effects of glucose ingestion also include significant decreases in total and free testosterone levels in men [11]. Notably, Chiu et al. (2014) [12] reported that SSB intake was inversely associated with progressive sperm motility, with lower sperm motility shown in adult males with higher intake of SSBs, but results of that study showed no significant association between SSB intake and testosterone levels.

Although animal studies have shown that SSB consumption has a negative impact on male fertility [13], human studies investigating relations between SSB intake and male reproductive hormone levels are lacking. Considering this lack, along with evidence that drinking SSBs correlates positively with male hypogonadism [1], we hypothesized that SSB consumption may decrease serum total testosterone concentrations in men 20 to 39 years old.

Thus, the purpose of the present study was to investigate whether consumption of SSBs is associated with lower serum total testosterone concentrations in men aged 20 to 39 years old by using data from the NHANES population-based survey that included validated physical examination measures, biological specimen collection, and clinical laboratory testing as measures of health status.

## Methods

### Data source

All data for this study were retrieved from the National Health and Nutrition Examination Survey (NHANES) 2011–2012 [14]. The NHANES database includes a stratified, multi-stage probability sample that is representative of the civilian non-institutionalized United States population (https://www.cdc.gov/Nchs/Nhanes/about_nhanes.htm). The NHANES survey was administered by the National Center for Health Statistics (NCHS) of the Centers for Disease Control and Prevention (CDC), and approved by its Institutional Review Board. The NCHS designed NHANES and collected the data after receiving informed consent from either the participants or their parents. Survey data have been collected continuously since 1999.

### Study population

The data of 991 male participants 20 to 39 years of age from NHANES 2011–2012 who had completed an interview and examination at a NHANES mobile examination center (MEC) were reviewed. Subjects who completed a dietary questionnaire/interview, body mass index (BMI) measurement, and a total serum testosterone measurement were eligible for inclusion. Although testosterone levels are known to peak at 55 to 60 years of age in healthy males [2], we specifically selected males 20 to 39 years old because reproductive activity is greatest during this period. Participants with incomplete information (missing dietary questionnaire/interview, BMI, or total testosterone test results) were excluded. Since the data of all participants are de-identified in the NHANES database, signed informed consent was not required.

### Main outcomes

The primary outcome for this study was serum testosterone level. Total testosterone levels were extracted from the NHANES Total Testosterone Section, and eligible participants were stratified based on serum testosterone level. Details regarding evaluation of testosterone levels are available at: https://www.cdc.gov/nchs/data/nhanes/nhanes_11_12/tst_g_met.pdf and https://wwwn.cdc.gov/Nchs/Nhanes/2011-2012/TST_G.htm. In brief, blood samples were collected once from each participant, and the timing of the blood collection was considered as serum testosterone levels exhibit a circadian rhythm. The NHANES laboratory examination protocol [15] states that serum testosterone was determined quantitatively using an electrochemiluminescence immunoassay on an Elecsys 2010 autoanalyzer (Roche Diagnostics, Indianapolis, IN, USA), as previously described [2]. Following recommendations of the International Society of Andrology (ISA), International Society for the Study of the Aging Male (ISSAM), European Association of Urology (EAU), European Academy of Andrology (EAA), and the American Society of Andrology (ASA) [16], the cut-off points of 231 ng/dL and 346 ng/dL testosterone (8 to 12 nmol/L) were considered as diagnostic of biochemical hypogonadism, as described previously [17]. Testosterone collection time was defined as *“session in which blood specimens were examined”*.

The main independent variable was SSB intake. The Dietary Interview – Individual Foods section of NHANES was used to estimate the SSB intake of each participant. In NHANES 2003–2004, two 24-h dietary recalls were available in the NHANES dietary interviewers guide and questionnaire [18], allowing NHANES 2003–2004 to provide information on participants’ intake of energy, nutrients, and specific food components and beverages. SSBs included all types of soda, fruit drinks, sport and energy drinks, sweetened coffees and teas, and other SSBs. To quantify the SSB consumption of each participant, total SSB consumption and kcal/day were calculated separately based on NHANES data (Dietary Interview Day 1) for each study subject. Then, the SSB (kcal/day) ratio was calculated and eligible male participants were classified into quartiles of SSBs (kcal/day) for analysis. Linear regression analysis was used to estimate the differences and 95% intervals (CI) in serum testosterone levels in higher quartiles (Q2 to Q4) of SSB intake (kcal/day) after adjusting for covariates and using male participants in the lowest quartile (Q1) as a reference.

### Variables

Other demographic and clinical variables examined as potential confounders included age, sex, race/ethnicity, poverty/income ratio, BMI, serum cotinine, heavy drinker status, and physical activity MET score. BMI (weight/height^2^) was measured by trained technicians at the NHANES MEC physical examination [19]. Obesity was defined as a BMI ≥30 kg/m^2^ according to the World Health Organization criteria [20]. Heavy drinker status was defined as respondents who reported consuming alcoholic beverages ≥4 times/week in response to the question *“In the past 12 months, how often did you drink any type of alcoholic beverage”?* Physical activity was estimated by summing the time spent weekly engaged in activities as reported by participants, multiplied by the metabolic equivalent of task (MET) value for that activity, which yields a MET-h index. One MET is the energy expenditure of 1 kcal/kg body weight per hours [21]. MET-min/week ≥500 was considered physically active, and < 500 MET-min/week was considered non-active.

#### Statistical analysis

Continuous variables are expressed as mean ± standard error, and categorical variables as unweighted counts (weighted %). Differences between categorical variables were examined using the chi-square test, and differences between continuous variables were determined using the Complex Samples General Linear Model (CSGLM) method. Univariate and multivariate logistic regression analyses were performed to explore associations between SSBs and low testosterone levels. Multivariate logistic regression was adjusted simultaneously for age, race, BMI, family income/poverty ratio, serum cotinine, heavy drinker status, and physical activity. All analyses included special sample weights (WTDRD1 [Dietary day-1, 2-year sample weight] for NHANES 2011–2012), stratum, and primary sampling units (PSU) per recommendations from the National Center for Health Statistics (NCHS), to address oversampling, non-response, non-coverage, and to provide nationally representative estimates. All statistical assessments were 2-sided and evaluated at the 0.05 level of significance. Statistical analyses were performed with IBM SPSS statistical software version 22 for Windows (IBM Corp., Armonk, NY, USA).

## Results

### Study sample

A total of 10,026 participants completed the NHANES MEC interview/ examination 2011–2012. Of these, there were 991 males 20–39 years old (mean, 28.9 ± 0.5 years). After excluding 322 participants without SSB intake information, 55 participants without testosterone level results, 3 participants without BMI information, and 66 participants who were missing other clinical information, the data of 545 participants were included in the analysis. Using the NHANES sample weight, the analytic sample size was equivalent to a population-based sample size of 25,563,095 participants.

### Subjects demographic and clinical characteristics

Demographic and clinical characteristics of the included subjects are summarized in Table 1. Among the 545 subjects, 486 (90.4%) had normal testosterone levels (defined as > 231 ng/dL) and 59 (9.6%) had low testosterone levels (≤231 ng/dL). The mean age of subjects with normal testosterone levels was 28.7 years, and 30.7 years for those with low testosterone levels. A significantly higher percentage of subjects with low testosterone levels had a BMI ≥25 kg/m^2^ as compared to those with normal testosterone levels (83.3% vs. 56.8%, *p* = 0.026) (Table 1). An analysis that evaluated the impact of blood sampling time with subject characteristics found sampling time did not statistically affect the findings (Additional file 1: Table S1).

**Table 1:** Subject demographic and clinical characteristics (Unweighted *n* = 545, Weighted *n* = 25,563,095)

	Total(n = 545)	Testosterone (ng/dL)		*p*-value
		Normal (≥231)(*n* = 486)	Low (< 231)(*n* = 59)	
Age (years)	28.9 ± 0.5	28.7 ± 0.5	30.7 ± 1.0	0.072
Race				0.597
Non-Hispanic White	213 (58.2)	184 (57.8)	29 (61.5)	
Mexican American	49 (9.2)	42 (8.7)	7 (13.6)	
Other Hispanic	64 (12.6)	59 (13.1)	5 (7.8)	
Non-Hispanic Black	124 (11.7)	112 (11.7)	12 (11.6)	
Other race	95 (8.3)	89 (8.7)	6 (5.4)	
Income/poverty ratio	2.6 ± 0.2	2.6 ± 0.2	2.2 ± 0.2	0.218
Serum cotinine (ng/mL)	62.7 ± 8.9	58.7 ± 8.4	100.5 ± 27.6	0.152
BMI				0.026^a^
< 25 kg/m^2^	204 (40.7)	199 (43.2)	5 (16.7)	
≥ 25 kg/m^2^	341 (59.3)	287 (56.8)	54 (83.3)	
Heavy drinker				0.110
No	497 (89.9)	440 (89.2)	57 (95.9)	
Yes	48 (10.1)	46 (10.8)	2 (4.1)	
Physical activity				0.245
≥ 500 MET-min/week	450 (86.5)	408 (87.0)	42 (81.7)	
< 500 MET-min/week	95 (13.5)	78 (13.0)	17 (18.3)	
SSBs (kcal/day)				0.510
Q1 (≤137.0)	137 (25.2)	129 (26.5)	8 (13.2)	
Q2 (138.0–272.0)	139 (26.6)	124 (26.4)	15 (28.1)	
Q3 (273.0–441.0)	133 (26.5)	119 (26.2)	14 (29.6)	
Q4 (442.0+)	136 (21.7)	114 (20.9)	22 (29.1)	

Continuous variables are shown as mean ± standard error; categorical variables are shown as unweighted counts (weighted %)

Abbreviations: BMI, body mass index (kg/m^2^); SSBs, sugar-sweetened beverages; MET, metabolic equivalent of task

^a^ Significant difference between normal and low testosterone levels, *p* < 0.05.

### Associations between SSBs and low testosterone level

Multivariate logistic regression analysis revealed that the odds of a low testosterone level was significantly increased with increasing SSB consumption (Q4 (442 kcal/day) vs. Q1 [≤137 kcal/day], adjusted odds ratio [aOR] = 2.29, *p* = 0.041) (Table 2). After adjusting for possible confounding variables, BMI was an independent risk factor for a low testosterone level, and subjects with a BMI ≥25 kg/m^2^ had a higher risk of having a low testosterone level than those with a BMI < 25 kg/m^2^ (aOR = 3.68, *p* = 0.044).

**Table 2:** Associations between SSBs and low testosterone levels

	Univariate		Multivariate	
	OR (95% CI)	p-value	aOR (95% CI)	p-value
SSBs (kcal/day)				
Q1 (≤137.0)	Reference		Reference	
Q2 (138.0–272.0)	2.12 (0.72,6.28)	0.162	1.96 (0.84,4.59)	0.111
Q3 (273.0–441.0)	2.26 (0.54,9.45)	0.246	2.20 (0.56,8.70)	0.244
Q4 (442.0+)	2.78 (1.04,7.45)	0.043*	2.29 (1.04,5.02)	0.041^a^
Age	1.06 (0.99,1.12)	0.075	1.04 (0.97,1.12)	0.221
Race				
Non-Hispanic White	Reference		Reference	
Mexican American	0.56 (0.13,2.51)	0.429	0.42 (0.10,1.81)	0.225
Other Hispanic	1.47 (0.41,5.28)	0.531	1.05 (0.28,3.93)	0.937
Non-Hispanic Black	0.93 (0.37,2.38)	0.881	0.80 (0.31,2.08)	0.635
Other race	0.59 (0.25,1.39)	0.207	0.61 (0.26,1.42)	0.233
Income/poverty ratio	0.87 (0.69,1.10)	0.238	0.89 (0.72,1.10)	0.274
Serum cotinine (ng/mL)	1.002 (0.9995,1.01)	0.095	1.002 (0.998,1.01)	0.353
BMI				
< 25 kg/m^2^	Reference		Reference	
≥ 25 kg/m^2^	3.80 (1.12,12.91)	0.034*	3.68 (1.04,13.01)	0.044^a^
Heavy drinker				
No	Reference		Reference	
Yes	0.35 (0.09,1.37)	0.124	0.40 (0.10,1.63)	0.187
Physical activity				
≥ 500 MET-min/week	Reference		Reference	
< 500 MET-min/week	1.50 (0.73,3.08)	0.247	1.19 (0.63,2.28)	0.572

Abbreviations: *BMI*, body mass index (kg/m^2^); *CI*, confidence interval; *OR*, odds ratio; *aOR*, adjusted odds ratio; *SSBs*, sugar-sweetened beverages; *MET*, metabolic equivalent of task

^a^ Significant factors, *p* < 0.05.

## Discussion

In the present study, which explored whether SSB consumption in adult males 20 to 39 years old in the United States decreased testosterone levels, multivariate analyses revealed that after adjusting for co-variates (age, race, family income, serum cotinine, BMI, heavy drinking, and physical activity), excessive consumption of SSBs (Q4 vs. Q1) was significantly associated with low testosterone levels. In addition, compared with subjects in Q1 (reference), subjects in Q2 and Q3 also had elevated odds of low testosterone levels, although these findings did not reach statistically significance. After adjusting for possible confounding variables, BMI was found to be an independent risk factor for low testosterone levels, and subjects with a BMI ≥25 kg/m^2^ had a higher risk of having a low testosterone level than did those with a BMI < 25 kg/m^2^.

The main findings of the present study are consistent with those of Chiu et al. [12] in terms of the influence of SSBs on male reproductive health, except that we focused on testosterone level rather than sperm motility. Chiu and colleagues reported that SSB intake was inversely associated with progressive sperm motility, but was not associated with other semen quality parameters or reproductive hormone levels, including testosterone [12]. In that study, men who consumed greater amounts of SSBs were more likely to have lower sperm motility. Similarly, in the present study, men who consumed SSBs had an increased risk of low testosterone levels, with the odds of low testosterone levels increasing with increasing SSB intake (kcal/day). Caronia et al. [11] showed more definitive results, demonstrating that oral glucose ingestion by adult males led to an abrupt drop in levels of total and free testosterone, although the authors cautioned that an immediate hormonal impact does not necessarily guarantee a cumulative effect.

Hypogonadism is associated with signs and symptoms that suggest testosterone deficiency, which may include low libido, erectile dysfunction, decreased muscle mass and strength, increased body fat, decreased bone mineral density/osteoporosis, and overall reductions in vitality and quality of life [22]. Testosterone levels are known to decline with aging, but can be decreased in adult males of any age, stressing the importance of defining and diagnosing hypogonadism. A report of the Canadian Men’s Health Foundation Multidisciplinary Guidelines Taskforce [23] focusing on the diagnosis and management of testosterone deficiency syndrome in men emphasized that in addition to clinical signs of testosterone deficiency, the diagnosis of testosterone deficiency requires documentation of testosterone levels below the local laboratory reference range. In the present study, hypogonadism was defined using the cut-off points recommended jointly by the ISA, ISSAM, EAU, EAA, and ASA [16], which have been applied in other studies. Garcia-Cruz et al. [17], for example, investigated adult testosterone levels and testosterone deficiency using the 2 recommended testosterone cut-off points of 231 ng/dL and 346 ng/dL as diagnostic for biochemical hypogonadism; a testosterone below 231 ng/dL was recognized as hypogonadism, and a result above 231 ng/dL was defined as normal. The testosterone levels that fell between 231 and 346 ng/dL constituted a “gray area,” and patients whose testosterone levels fell within that gray area were advised to receive a thorough examination to establish a final diagnosis of hypogonadism [23]. Recommendations of the professional associations listed above advise that men with total testosterone levels below 230 ng/dL would benefit from testosterone treatment [11]. For example, in a study that evaluated the roles of testosterone and sildenafil on sleep-related erections, all hypogonadal subjects had very low testosterone levels of < 200 ng/dL, and sleep-related erections were found to improve with the use of sildenafil [24].

In the present study, after adjusting for SSB intake (kcal/day), age, race, family income, serum cotinine, heavy drinker status, and physical activity, BMI was significantly associated with low testosterone levels, which confirms findings of previous reports, but was not a primary finding of this study. However, SSB intake is strongly associated with obesity [25], and obesity is associated with semen quality [26]. Chiu et al. [12] did not find evidence of mediation by BMI, finding instead that the association between SSBs and sperm motility was observed more among lean men than overweight or obese men.

A review of dietary patterns and male fertility parameters found that a healthy diet with sufficient omega-3 fatty acids, antioxidants, and vitamins that were also low in saturated and total fatty acids was inversely associated with indicators of low semen quality, and diets high in processed foods, full-fat diary, and SSBs were inversely associated with semen quality [27]. Indeed, SSBs are associated with weight gain and obesity [28], metabolic syndrome [6], and diabetes [29], but it is not known if semen quality is related to glucose and insulin, or to numerous possible contaminants such as phthalates and other substances leached from the plastic containers that hold the beverages [30]. Our hypothesis considered that some component in the sugary beverages may decrease the serum level of testosterone in adult males20 to 39 years old, but we did not investigate the relation with glucose, and have not concluded that the causative component may be glucose. The mechanisms explaining the associations of diet and sperm function and fertility remain unknown, although sperm cells are known to require glucose for proper functioning [31]. Further investigation is needed to determine whether the SSB-induced changes in testosterone levels is associated with glucose and insulin, phthalates, or other substances in the beverages or their containers.

### Strengths and limitations

A strength of the present study is the use of NHANES 2011–2012 data. NHANES is an ongoing national health survey conducted in the United States, and includes comprehensive demographic information, validated physical examination measures such as anthropometrics and blood pressure conducted by trained technicians, biological specimen collection and clinical laboratory studies, and other valid measures of health status. Data from the NHANES cycles (1999 to 2015) are drawn from a large, diverse, nationally representative sample of the United States population, making our findings generalizable to the overall population of the country, although it is unclear if the findings are generalizable to other populations. Since the study was a retrospective analysis of data from the cross-sectional NHANES survey, it is not possible to determine any causality or identify putative mediators. Another limitation is that dietary intake data were derived from 24-h dietary recall interviews that may be affected by participants’ inaccurate recall or reporting, resulting in possible recall bias. Additional prospective studies are needed to expand the investigation of the effects of SSBs on testosterone levels in adult males, and to assess the possible underlying mechanisms.

## Conclusions

Consumption of SSBs is significantly associated with low serum testosterone levels in men 20–39 years old. The effects of SSB consumption on testosterone levels in adult males must be considered if primary and secondary hypogonadism have been ruled out as a source of low testosterone and related symptoms.

## Additional file

Additional file 1: **Table S1.** Distribution and difference between testosterone level and collection time. (DOCX 14 kb).
