# Supplementary material for: Sugar-sweetened beverage intake and serum testosterone levels in adult males 20–39 years old in the United States
Source: Reprod Biol Endocrinol. 2018 Jun 23;16:61. doi: 10.1186/s12958-018-0378-2 (PMC6015465; doi:10.1186/s12958-018-0378-2)
Supplement: Supplementary file 1 — Table S1. Distribution and difference between testosterone level and collection time. (DOCX 14 kb). [file 12958_2018_378_MOESM1_ESM.docx]

Additional file 1: Table S1 Distribution and difference between testosterone level and collection time

|  | Testosterone (ng/dL) | | | | |
| --- | --- | --- | --- | --- | --- |
|  | Continuous | | Categorical | | |
|  | Mean ± SE | p-value | Normal (≥ 231) | Low (< 231) | p-value |
| Collection time |  | 0.080 |  | | 0.204 |
| Morning (n=281) | 474.6 ± 21.7 |  | 262 (50.9) | 19 (34.5) |  |
| Afternoon (n=173) | 419.5 ± 20.4 |  | 146 (27.4) | 27 (43.6) |  |
| Evening (n=91) | 414.3 ± 14.5 |  | 78 (21.7) | 13 (21.8) |  |

Continuous variables are expressed as mean ± standard error, and tested by complex samples general linear regression model; categorical variables as unweighted counts (weighted %), and tested by complex samples crosstabs.

SE, standard error.
